# Supplementary material for: hkb is required for DIP-α expression and target recognition in the Drosophila neuromuscular circuit
Source: Commun Biol. 2024 Apr 27;7:507. doi: 10.1038/s42003-024-06184-8 (PMC11055905; doi:10.1038/s42003-024-06184-8)
Supplement: Supplementary file 2 — Supplementary Information [file 42003_2024_6184_MOESM2_ESM.pdf]

Supplementary Table 1 All Df lines and subscreen lines used in this study.

BL Number or targeted gene	Genotype
1842	*Df(3R)Antp17/TM3, Sb[1] Ser[1]*
1990	*Df(3R)Tpl10, Dp(3;3)Dfd[rv1], kni[ri-1] Dfd[rv1] p[p] Doa[10]/TM3, Sb[1]*
2352	*Df(3R)X3F, P{ry[+t7.2]=RP49}mtg[P2] e[1]/MKRS*
2596	*Df(3L)6B-29+Df(3R)6B-29, kni[ri-1] p[p]/TM3, Ser[1]*
2597	*Df(3R)10-65, kni[ri-1] p[p]/TM3, Ser[1]*
3486	*Df(3R)Ubx109/Dp(3;3)P5*
6962	*w[1118]; Df(3R)ED2, P{w[+mW.Scer\FRT.hs3]=3'.RS5+3.3'}koko[ED2]/TM6C, cu[1] Sb[1]*
7443	*Df(3R)BSC47, st[1] ca[1]/TM3, P{w[+m*]=Ubx-lacZ.w[+]}TM3, Sb[1]*
8029	*w[1118]; Df(3R)ED5577, P{w[+mW.Scer\FRT.hs3]=3'.RS5+3.3'}ED5577/TM6C, cu[1] Sb[1]*
8103	*w[1118]; Df(3R)ED5177, P{w[+mW.Scer\FRT.hs3]=3'.RS5+3.3'}ED5177/TM6C, cu[1] Sb[1]*
8105	*w[1118]; Df(3R)ED6232, P{w[+mW.Scer\FRT.hs3]=3'.RS5+3.3'}ED6232/TM6C, cu[1] Sb[1]*
8684	*w[1118]; Df(3R)ED6096, P{w[+mW.Scer\FRT.hs3]=3'.RS5+3.3'}ED6096/TM6C, cu[1] Sb[1]*
8685	*w[1118]; Df(3R)ED7665, P{w[+mW.Scer\FRT.hs3]=3'.RS5+3.3'}ED7665/TM6C, cu[1] Sb[1]*
8957	*w[1118]; Df(3R)ED5514, P{w[+mW.Scer\FRT.hs3]=3'.RS5+3.3'}ED5514/TM6C, cu[1] Sb[1]*
8964	*w[1118]; Df(3R)ED6025, P{w[+mW.Scer\FRT.hs3]=3'.RS5+3.3'}ED6025/TM6C, cu[1] Sb[1]*
8965	*w[1118]; Df(3R)ED5156, P{w[+mW.Scer\FRT.hs3]=3'.RS5+3.3'}ED5156/TM6C, cu[1] Sb[1]*
9077	*w[1118]; Df(3R)ED5330, P{w[+mW.Scer\FRT.hs3]=3'.RS5+3.3'}ED5330/TM6C, cu[1] Sb[1]*
9082	*w[1118]; Df(3R)ED5474, P{w[+mW.Scer\FRT.hs3]=3'.RS5+3.3'}ED5474/TM6C, cu[1] Sb[1]*
9084	*w[1118]; Df(3R)ED5518, P{w[+mW.Scer\FRT.hs3]=3'.RS5+3.3'}ED5518/TM6C, cu[1] Sb[1]*
9090	*w[1118]; Df(3R)ED5644, P{w[+mW.Scer\FRT.hs3]=3'.RS5+3.3'}ED5644/TM6C, cu[1] Sb[1]*
9204	*w[1118]; Df(3R)ED5339, P{w[+mW.Scer\FRT.hs3]=3'.RS5+3.3'}ED5339/TM6C, cu[1] Sb[1]*
9210	*w[1118]; Df(3R)ED6255, P{w[+mW.Scer\FRT.hs3]=3'.RS5+3.3'}ED6255/TM6C, cu[1] Sb[1]*
9211	*w[1118]; Df(3R)ED6220, P{w[+mW.Scer\FRT.hs3]=3'.RS5+3.3'}ED6220/TM6C, cu[1] Sb[1]*
9226	*w[1118]; Df(3R)ED5100, P{w[+mW.Scer\FRT.hs3]=3'.RS5+3.3'}ED5100/TM6C, cu[1] Sb[1]*

9227	*w[1118]; Df(3R)ED5428, P{w[+mW.Scer\FRT.hs3]=3'.RS5+3.3'}ED5428/TM6C, cu[1] Sb[1]*
9481	*w[1118]; Df(3R)ED10639, P{w[+mW.Scer\FRT.hs3]=3'.RS5+3.3'}ED10639/TM6C, cu[1] Sb[1]*
9482	*w[1118]; Df(3R)ED10642, P{w[+mW.Scer\FRT.hs3]=3'.RS5+3.3'}ED10642/TM6C, cu[1] Sb[1]*
9487	*w[1118]; Df(3R)ED10845, P{w[+mW.Scer\FRT.hs3]=3'.RS5+3.3'}ED10845/TM6C, cu[1] Sb[1]*
24137	*w[1118]; Df(3R)ED5664, P{w[+mW.Scer\FRT.hs3]=3'.RS5+3.3'}ED5664/TM6C, cu[1] Sb[1]*
24139	*w[1118]; Df(3R)ED5938, P{w[+mW.Scer\FRT.hs3]=3'.RS5+3.3'}ED5938/TM6C, cu[1] Sb[1]*
24142	*w[1118]; Df(3R)ED6346, P{w[+mW.Scer\FRT.hs3]=3'.RS5+3.3'}ED6346/TM6C, cu[1] Sb[1]*
24143	*w[1118]; Df(3R)ED6361, P{w[+mW.Scer\FRT.hs3]=3'.RS5+3.3'}ED6361/TM6C, cu[1] Sb[1]*
24516	*w[1118]; Df(3R)ED50003, P{w[+mW.Scer\FRT.hs3]=3'.RS5+3.3'}ED50003/TM6C, cu[1] Sb[1]*
24909	*w[1118]; Df(3R)BSC321/TM6C, Sb[1] cu[1]*
24965	*w[1118]; Df(3R)BSC461/TM6C, Sb[1] cu[1]*
24968	*w[1118]; Df(3R)BSC464/TM6C, Sb[1] cu[1]*
24970	*w[1118]; Df(3R)BSC466/TM6C, Sb[1] cu[1]*
24971	*w[1118]; Df(3R)BSC467/TM6C, Sb[1] cu[1]*
24973	*w[1118]; Df(3R)BSC469/TM6C, Sb[1] cu[1]*
24980	*w[1118]; Df(3R)BSC476/TM6C, Sb[1] cu[1]*
24983	*w[1118]; Df(3R)BSC479/TM6C, Sb[1] cu[1]*
24990	*w[1118]; Df(3R)BSC486/TM6C, Sb[1] cu[1]*
24993	*w[1118]; Df(3R)BSC489/TM6C, Sb[1] cu[1]*
25001	*w[1118]; Df(3R)BSC497/TM6C, Sb[1] cu[1]*
25005	*w[1118]; Df(3R)BSC501/TM6C, Sb[1] cu[1]*
25006	*w[1118]; Df(3R)BSC502/TM6C, Sb[1] cu[1]*
25007	*w[1118]; Df(3R)BSC503/TM6C, Sb[1] cu[1]*
25008	*w[1118]; Df(3R)BSC504/TM6C, Sb[1] cu[1]*
25011	*w[1118]; Df(3R)BSC507/TM6C, Sb[1] cu[1]*
25019	*w[1118]; Df(3R)BSC515/TM6C, Sb[1] cu[1]*
25075	*w[1118]; Df(3R)BSC547/TM6C, Sb[1]*
25077	*w[1118]; Df(3R)BSC549/TM6C, Sb[1]*
25390	*w[1118]; Df(3R)BSC567/TM6C, Sb[1]*
25694	*w[1118]; Df(3R)BSC619/TM6C, cu[1] Sb[1]*
25695	*w[1118]; Df(3R)BSC620/TM6C, cu[1] Sb[1]*
25696	*w[1118]; Df(3R)BSC621/TM6C, cu[1] Sb[1]*
25724	*w[1118]; Df(3R)BSC633/TM6C, cu[1] Sb[1]*
25740	*w[1118]; Df(3R)BSC650/TM6C, Sb[1] cu[1]*

26529	*w[1118]; Df(3R)BSC677, P+PBac{w[+mC]=XP3.WH3}BSC677/TM6C, Sb[1] cu[1]*
26533	*w[1118]; Df(3R)BSC681, P+PBac{w[+mC]=XP3.RB5}BSC681/TM6C, Sb[1] cu[1]*
26580	*w[1118]; Df(3R)BSC728, P+PBac{w[+mC]=XP3.RB5}BSC728/TM6C, Sb[1] cu[1]*
26836	*w[1118]; Df(3R)BSC738/TM6C, Sb[1] cu[1]*
26839	*w[1118]; Df(3R)BSC741/TM6C, Sb[1] cu[1]*
26846	*w[1118]; Df(3R)BSC748, P+PBac{w[+mC]=XP3.WH3}BSC748/TM6C, Sb[1] cu[1]*
26847	*w[1118]; Df(3R)BSC749, P+PBac{w[+mC]=XP3.WH3}BSC749/TM6C, Sb[1] cu[1]*
26848	*w[1118]; Df(3R)BSC750/TM6C, Sb[1] cu[1]*
27365	*w[1118]; Df(3R)BSC793/TM6C, Sb[1] cu[1]*
27404	*w[1118]; Df(3R)FDD-0317950/TM6C, Sb[1] cu[1]*
27580	*w[1118]; Df(3R)BSC819, P+PBac{w[+mC]=XP3.RB5}BSC819/TM6C, Sb[1] cu[1]*
29667	*w[1118]; Df(3R)ED6280, P{w[+mW.Scer\FRT.hs3]=3'.RS5+3.3'}ED6280/TM6C, cu[1] Sb[1]*
7633	*w[1118]; Df(3R)Exel6154, P{w[+mC]=XP-U}Exel6154/TM6B, Tb[1]*
7634	*w[1118]; Df(3R)Exel6155, P{w[+mC]=XP-U}Exel6155/TM6B, Tb[1]*
7638	*w[1118]; Df(3R)Exel6159, P{w[+mC]=XP-U}Exel6159/TM6B, Tb[1]*
7675	*w[1118]; Df(3R)Exel6196, P{w[+mC]=XP-U}Exel6196/TM6B, Tb[1]*
7676	*w[1118]; Df(3R)Exel6197, P{w[+mC]=XP-U}Exel6197/TM6B, Tb[1]*
7680	*w[1118]; Df(3R)Exel6201, P{w[+mC]=XP-U}Exel6201/TM6B, Tb[1]*
7681	*w[1118]; Df(3R)Exel6202, P{w[+mC]=XP-U}Exel6202/TM6B, Tb[1]*
7682	*w[1118]; Df(3R)Exel6203, P{w[+mC]=XP-U}Exel6203/TM6B, Tb[+]*
7692	*w[1118]; Df(3R)Exel6214, P{w[+mC]=XP-U}Exel6214/TM6B, Tb[1]*
7731	*w[1118]; Df(3R)Exel6264, P{w[+mC]=XP-U}Exel6264/TM6B, Tb[+]*
7737	*w[1118]; Df(3R)Exel6270, P{w[+mC]=XP-U}Exel6270/TM6B, Tb[1]*
7739	*w[1118]; Df(3R)Exel6272, P{w[+mC]=XP-U}Exel6272/TM6B, Tb[1]*
7997	*w[1118]; Df(3R)Exel7378/TM6B, Tb[1]*
9497	*w[1118]; Df(3R)BSC137/TM6B, Tb[+]*
9500	*w[1118]; Df(3R)BSC140/TM6B, Tb[+]*
9501	*w[1118]; Df(3R)BSC141/TM6B, Tb[+]*
8923	*w[1118]; Df(3R)ED6085, P{w[+mW.Scer\FRT.hs3]=3'.RS5+3.3'}ED6085/TM2*
7413	*Df(3R)BSC43, st[1] ca[1]/TM2, p[p]*
8104	*w[1118]; Df(3R)ED5780, P{w[+mW.Scer\FRT.hs3]=3'.RS5+3.3'}ED5780/TM2*
9208	*w[1118]; Df(3R)ED5815, P{w[+mW.Scer\FRT.hs3]=3'.RS5+3.3'}ED5815/TM2*
25021	*w[1118]; Df(3R)BSC517/TM2*
37537	*w[1118]; Df(3R)ED5623, P{w[+mW.Scer\FRT.hs3]=3'.RS5+3.3'}ED5623/TM2*
8967	*y[*] w[1118]/Dp(1;Y)y[+]; Df(3R)ED5147, P{w[+mW.Scer\FRT.hs3]=3'.RS5+3.3'}ED5147/TM6C, cu[1] Sb[1]*

1467	*Dp(3;1)P115/+; Df(3R)P115, e[11]/TM1, Sb*
2155	*Df(3R)A113/In(3R)C, Sb[1] cd[1] Tb[1] ca[1]; Dp(3;1)34*
2234	*Df(3R)R133, B[S]/TM3, Sb[1]; Dp(3;1)124P*
3547	*Df(3R)L127/TM6; Dp(3;1)B152*
6367	*Df(3R)slo3/MKRS; Dp(3;2)slo3/+*
auxilin	*w[*]; aux[D128]/TM6B, P{w[+mW.hs]=Ubi-GFP.S65T}PAD2, Tb[1]*
abstrakt	*P{ry[+t7.2]=PZ}abs[00620] ry[506]/TM3, ry[RK] Sb[1] Ser[1]*
complexin	*y[1] w[*]; Mi{y[+mDint2]=MIC}cpx[MI00784]/TM3, Sb[1] Ser[1]*
vps24	*y[1] w[67c23]; P{w[+mC] y[+mDint2]=EPgy2}Vps24[EY04708]/TM3, Sb[1] Ser[1]*
huckebein	*hkb[2]/TM3, Sb[1] Ser[1]*
contactin	*w[1118]; P{w[+mC]=EP}Cont[G5080]/TM6C, Sb[1]*
tube	*st[1] tub[2] e[1]/TM8, l(3)DTS4[1]*
lost	*y[1] w[67c23]; P{w[+mC] y[+mDint2]=EPgy2}lost[EY11645]*
