# Supplementary material for: hkb is required for DIP-α expression and target recognition in the Drosophila neuromuscular circuit
Source: Commun Biol. 2024 Apr 27;7:507. doi: 10.1038/s42003-024-06184-8 (PMC11055905; doi:10.1038/s42003-024-06184-8)
Supplement: Supplementary file 3 — Description of Additional Supplementary Files [file 42003_2024_6184_MOESM3_ESM.pdf]

Description of Additional Supplementary Files

File name: Supplementary Data 1

Description: The source data of the graphs in this study.
