# Supplementary material for: hkb is required for DIP-α expression and target recognition in the Drosophila neuromuscular circuit
Source: Commun Biol. 2024 Apr 27;7:507. doi: 10.1038/s42003-024-06184-8 (PMC11055905; doi:10.1038/s42003-024-06184-8)
Supplement: Supplementary file 5 — Reporting Summary [file 42003_2024_6184_MOESM5_ESM.pdf]

nature portfolio

Corresponding author(s): Carrillo R.A. and Wang Y.

Last updated by author(s): Mar 25, 2024

Reporting Summary

Nature Portfolio wishes to improve the reproducibility of the work that we publish. This form provides structure for consistency and transparency in reporting. For further information on Nature Portfolio policies, see our Editorial Policies and the Editorial Policy Checklist.

Statistics

For all statistical analyses, confirm that the following items are present in the figure legend, table legend, main text, or Methods section.

n/a	Confirmed	
☐	☒	The exact sample size (*n*) for each experimental group/condition, given as a discrete number and unit of measurement
☐	☒	A statement on whether measurements were taken from distinct samples or whether the same sample was measured repeatedly
☐	☒	The statistical test(s) used AND whether they are one- or two-sided<br>*Only common tests should be described solely by name; describe more complex techniques in the Methods section.*
☐	☒	A description of all covariates tested
☐	☒	A description of any assumptions or corrections, such as tests of normality and adjustment for multiple comparisons
☐	☒	A full description of the statistical parameters including central tendency (e.g. means) or other basic estimates (e.g. regression coefficient) AND variation (e.g. standard deviation) or associated estimates of uncertainty (e.g. confidence intervals)
☐	☒	For null hypothesis testing, the test statistic (e.g. *F*, *t*, *r*) with confidence intervals, effect sizes, degrees of freedom and *P* value noted<br>*Give P values as exact values whenever suitable.*
☒	☐	For Bayesian analysis, information on the choice of priors and Markov chain Monte Carlo settings
☒	☐	For hierarchical and complex designs, identification of the appropriate level for tests and full reporting of outcomes
☒	☐	Estimates of effect sizes (e.g. Cohen's *d*, Pearson's *r*), indicating how they were calculated

Our web collection on statistics for biologists contains articles on many of the points above.

Software and code

Policy information about availability of computer code

Data collection	Representative images were acquired on a Zeiss LSM800 confocal microscope using a 40X plan-neofluar 1.3 NA objective, or a 63X plan-apo 1.4 NA objective. The same imaging parameters were applied to samples from the same set of experiments. To quantify innervation frequency, samples were visualized under a Zeiss AxioImager M2 scope with a Lumen light engine with a 20× Plan Apo 0.8 NA objective or an Olympus BX43 with an X-Cite 120LEDmini LED fluorescent illuminator.
Data analysis	Images were analyzed and processed in ImageJ.

For manuscripts utilizing custom algorithms or software that are central to the research but not yet described in published literature, software must be made available to editors and reviewers. We strongly encourage code deposition in a community repository (e.g. GitHub). See the Nature Portfolio guidelines for submitting code & software for further information.

Data

Policy information about availability of data

All manuscripts must include a data availability statement. This statement should provide the following information, where applicable:

- Accession codes, unique identifiers, or web links for publicly available datasets
- A description of any restrictions on data availability
- For clinical datasets or third party data, please ensure that the statement adheres to our policy

Original data is available upon request.

Research involving human participants, their data, or biological material

Policy information about studies with human participants or human data. See also policy information about sex, gender (identity/presentation), and sexual orientation and race, ethnicity and racism.

Reporting on sex and gender	This study did not include human samples.
Reporting on race, ethnicity, or other socially relevant groupings	This study did not include human samples.
Population characteristics	This study did not include human samples.
Recruitment	This study did not include human samples.
Ethics oversight	This study did not include human samples.

Note that full information on the approval of the study protocol must also be provided in the manuscript.

Field-specific reporting

Please select the one below that is the best fit for your research. If you are not sure, read the appropriate sections before making your selection.

☒ Life sciences ☐ Behavioural & social sciences ☐ Ecological, evolutionary & environmental sciences

For a reference copy of the document with all sections, see nature.com/documents/nr-reporting-summary-flat.pdf

Life sciences study design

All studies must disclose on these points even when the disclosure is negative.

Sample size	Sample sizes were determined by existing studies in the field to enable statistical analyses and reproducibility. In general, to quantify innervation frequency, at least 6 animals/29 NMJs were examined. For DIP-alpha expression in VNC, at least 6 embryos of given genotype were examined.
Data exclusions	No data were excluded.
Replication	All experiments were performed with at least two independent biological replicates and data points were pulled and analyzed together. All attempts at replication were successful.
Randomization	Male and female flies were collected and randomly assigned into different cross. Female larvae were randomly selected for recording.
Blinding	Experimenters were blinded in genetics screening as we did not know the genes covered in each deficiency. In following validation, experimenters were blinded when counting innervation frequency, and the innervation frequencies were linked with genotypes after counting.

Reporting for specific materials, systems and methods

We require information from authors about some types of materials, experimental systems and methods used in many studies. Here, indicate whether each material, system or method listed is relevant to your study. If you are not sure if a list item applies to your research, read the appropriate section before selecting a response.

Materials & experimental systems

n/a	Involved in the study	
☐	☒	Antibodies
☒	☐	Eukaryotic cell lines
☒	☐	Palaeontology and archaeology
☐	☒	Animals and other organisms
☒	☐	Clinical data
☒	☐	Dual use research of concern
☒	☐	Plants

Methods

n/a	Involved in the study	
☒	☐	ChIP-seq
☒	☐	Flow cytometry
☒	☐	MRI-based neuroimaging

Antibodies

Antibodies used

Rabbit anti-GFP, Michael Glotzer Lab, University of Chicago, 1:40,000
Rabbit anti-Eve, Ellie Heckscher Lab, University of Chicago, 1:1000
Mouse anti-DLG, Developmental Studies Hybridoma Bank, #4F3, 1:100
Mouse anti-FasII, Developmental Studies Hybridoma Bank, #1D4, 1:100
Goat anti-rabbit-Alexa Fluor 488, Thermo Fisher Scientific, #A11008, 1:500
Goat anti-rabbit-Alexa Fluor 568, Thermo Fisher Scientific, #A11036, 1:500
Goat anti-mouse-Alexa Fluor 568, Thermo Fisher Scientific, #A11031, 1:500
Goat anti-mouse-Alexa Fluor 647, Thermo Fisher Scientific, #A32728, 1:500
Goat anti-HRP-Alexa Fluor 405, Jackson ImmunoResearch, #123-475-021, 1:100
Goat anti-HRP-Alexa Fluor 647, Jackson ImmunoResearch, #123-605-021, 1:100

Validation

Rabbit anti-GFP, Michael Glotzer Lab, University of Chicago
Rabbit anti-Eve, Ellie Heckscher Lab, University of Chicago
Mouse anti-DLG, Developmental Studies Hybridoma Bank, #4F3
Mouse anti-FasII, Developmental Studies Hybridoma Bank, #1D4
Goat anti-rabbit-Alexa Fluor 488, Thermo Fisher Scientific, #A11008
Goat anti-rabbit-Alexa Fluor 568, Thermo Fisher Scientific, #A11036
Goat anti-mouse-Alexa Fluor 568, Thermo Fisher Scientific, #A11031
Goat anti-mouse-Alexa Fluor 647, Thermo Fisher Scientific, #A32728
Goat anti-HRP-Alexa Fluor 405, Jackson ImmunoResearch, #123-475-021
Goat anti-HRP-Alexa Fluor 647, Jackson ImmunoResearch, #123-605-021

Animals and other research organisms

Policy information about studies involving animals; ARRIVE guidelines recommended for reporting animal research, and Sex and Gender in Research

Laboratory animals

w1118 (Carrillo et al., 2015)
DIP-α-GAL4 (Ashley et al., 2019)
UAS-2×EGFP
dpr10CR (Xu et al., 2018)
DIP-αCR (Xu et al., 2018)
DIP-α-EGFP (Tan et al., 2015)
hkbA321R1 (BL#2059) (Gaul and Weigel, 1990)
hkb2 (BL#5457) (Bossing et al., 1996)
eveΔRN2(Fujioka et al., 2003)
Df lines discussed in this paper are: ED5100 (BL#9226), ED5046 (BL#9197), ED5142 (BL#9198).
All lines used for screen and sub-screen are listed in Table 1 (Cook et al., 2012; Roote and Russell, 2012).

Wild animals

No wild animals were used in the study.

Reporting on sex

In our deficiency screen and following trans-heterozygotes assay where DIP-alpha is involved, only female larvae were collected and examined, because DIP-alpha is located on the first chromosome and we need to examine trans-heterozygotes. In other experiments including DIP-alpha-EGFP expression and genetic interaction assay between hkb and eve, both male and female larvae were examined.

Field-collected samples

This study did not include samples collected from the field.

Ethics oversight

No ethical approval is required for this study.

Note that full information on the approval of the study protocol must also be provided in the manuscript.
